# Deep‐Learning‐Based Image Reconstruction to Improve End‐Diastolic and Systolic Cardiac T1 Mapping

**DOI:** 10.1002/mrm.70353

**Published:** 2026-03-19

**Authors:** Daniel Amsel, Jens Wetzl, Daniel Giese, Christoph Tillmanns, Rolf Gebker, Kelvin Chow, Michaela Schmidt, Andreas Lingg, Jens Kübler, Patrick Krumm, Thomas Küstner

**Affiliations:** ^1^ Medical Image and Data Analysis (MIDAS.lab), Department of Diagnostic and Interventional Radiology University of Tuebingen Tuebingen Germany; ^2^ Research & Clinical Translation, Magnetic Resonance, Siemens Healthineers AG Erlangen Germany; ^3^ Diagnostikum Berlin Germany; ^4^ Cardiovascular MR R&D, Siemens Healthcare Ltd Calgary Canada; ^5^ Department of Radiology University Hospital Tuebingen Tuebingen Germany

**Keywords:** deep learning reconstruction, myocardial T1 mapping, quantitative MRI

## Abstract

**Purpose:**

To develop an image reconstruction method that enables increased spatial resolution cardiac T1 mapping in both the end‐diastolic and systolic phase, that shows high T1 agreement with the clinical standard. The resolution gain is achieved by increasing the acceleration rate of MOLLI single‐shot images to *R* = 4, while maintaining a sufficiently short acquisition window.

**Methods:**

A modified end‐to‐end variational network (MappingVN) is proposed. The modifications include a re‐ordered sheared‐grid sampling pattern, 2D + contrast convolutions and the use of patchwise squeeze‐and‐excitation layers. The method was evaluated in terms of image quality and T1 agreement with reference MOLLI T1 maps using retrospectively undersampled patient data. Furthermore, the method was additionally evaluated in a prospective setting comparing high‐resolution T1 maps (1.14 × 1.14 mm^2^) to reference T1 maps in standard resolution (1.41 × 2.13 mm^2^). Finally, the applicability for systolic T1 mapping was explored using increased acceleration to shorten the acquisition window.

**Results:**

The MappingVN showed improved SSIM scores of 0.95 and 0.98 on 1.5T and 3 T compared to 0.93 and 0.96 for GRAPPA. In high‐resolution end‐diastolic T1 maps stronger T1 agreement (MappingVN: −3 ± 69 ms on 1.5T, −11 ± 70 ms on 3T, GRPPA: −9 ± 129 ms on 1.5T, 14 ± 106 ms) could be observed. For systolic T1 mapping the MappingVN reduced the occurrence of motion artifacts.

**Conclusion:**

The proposed method enables high spatial resolution cardiac T1 mapping in both end‐diastolic and systolic phases. Resulting maps show good T1 agreement with the clinical standard and may improve the visibility of small focal lesions while reducing partial volume effects.

## 1 Introduction

Cardiac T1 mapping is a quantitative magnetic resonance imaging (MRI) technique that has seen increasing utilization in clinical practice [1, 2]. Resulting T1 maps hold valuable information about given tissue properties that can be used to diagnose conditions like diffuse fibrosis and cardiomyopathies [1, 3]. Multiple cardiac T1 mapping sequences exist [4, 5, 6], including the modified look‐locker inversion recovery sequence (MOLLI) [7] which is widely used in clinical practice for its high reproducibility and precision [8, 9]. It acquires single‐shot images following varying delays after inversion pulses and includes waiting times to allow relaxation. Single‐shot images are typically acquired in diastole during which the motion of the heart is minimized. Particularly in patients with high heart rates or arrythmias [10, 11], this quiescent phase can be very short and motion artifacts can occur. On the other hand, higher spatial resolutions are of interest for patients with thinner myocardial walls [8] or the improved visualization and characterization of small lesions such as scars [12], while systolic imaging may improve robustness of the acquisition in the presence of arrythmias [10, 11]. Increasing the spatial resolution of cardiac T1 maps would therefore address important unmet clinical needs. Super resolution algorithms [13, 14, 15] on T1 maps have been proposed mainly enhancing resolution in slice direction [16, 17]. Another possibility is to acquire the data in higher resolution while simultaneously utilizing accelerated imaging techniques. Parallel imaging techniques such as GRAPPA [18] or SENSE [19] are used in clinical T1 mapping protocols. Alternatively, random undersampling paired with a compressed sensing (CS) reconstruction [20, 21] can also accelerate inversion recovery acquisitions [22, 23], as shown for example by Nishigake et al. [24], who applied CS with *R* = 3.2 to increase the spatial resolution of cardiac T1 maps from 2 to 1.4 mm^2^. Cardiac images exhibit a low‐rank property that can be exploited in low‐rank‐based methods [25], as for example shown by Gao et al. [26], who achieved a mapping resolution of 1.25 mm^2^ by reconstructing *R* = 16 accelerated radial MOLLI acquisitions.

In recent years, several deep‐learning‐based image reconstruction methods were developed for various imaging tasks [27, 28], including T1 mapping [27, 29, 30]. Many of these approaches reconstruct quantitative maps directly from undersampled *k*‐space data [31, 32, 33], allowing the physical relaxation process to be incorporated in the reconstruction. Additionally, they leverage recurrence [31] or low‐rank‐based approaches [34] to exploit the redundant information across the contrasts. One example is the MyoMapNet [35] which focuses on accelerating cardiac T1 mapping while keeping the same resolution. Despite showing good agreement with conventional reconstruction pipelines for quantitative imaging, some of these methods lack explainability of intermediate steps.

Next to developing full mapping pipelines, recent works have also focused on reconstructing the inversion recovery images from accelerated MOLLI acquisitions, with research interest for this topic being influenced by the MICCAI CMRxRecon Challenge [36]. Respective algorithms range from artifact removal in image space using convolutional neural networks [37] to utilizing unrolled networks like MoDL [38] or the end‐to‐end Variational Network (E2E VarNet) [39]. Further trends in the domain of cardiac T1 mapping reconstruction include unrolled networks that are modified toward MOLLI acquisitions by using different network layer configurations to exploit redundancies across the contrast dimension. Recent literature utilizes attention mechanisms in the form of channel attention [40, 41] or transformer layers [42, 43, 44]. Also, low rank‐based approaches [45] and relaxometry‐based constraints [46] are applied. While these methods demonstrate strong performance in reconstructing inversion recovery images retrospectively, they often lack evaluation of T1 accuracy and validation with prospectively acquired high‐resolution MOLLI data.

The influence of the sampling pattern on deep learning‐based image reconstruction was previously investigated [47]. These studies include *k*–*t* reconstruction techniques that utilize sheared‐grid sampling patterns in combination with data sharing layers [48, 49, 50]. While these works highlight the beneficial interaction between sheared‐grid patterns and specific network components, they only investigated the reconstruction of Cine [49, 50] and DCE [48] data with minimal contrast changes. Also, joint optimization of the reconstruction and sampling strategy was explored [51]. Nevertheless, these aspects have not yet been studied in the context of deep learning‐based image reconstruction for cardiac T1 mapping.

In this work, we propose a deep learning‐based approach for reconstructing inversion recovery images from accelerated (*R* = 4) MOLLI acquisitions with high spatial resolution, labeled MappingVN. The MappingVN is based on an unrolled E2E VarNet that was adapted for the reconstruction of MOLLI acquisitions. Compared to previous works, we choose a combination of convolution and channel attention layers by extending the classical Squeeze‐and‐Excitation [52] to patchwise Squeeze‐and‐Excitation (pSE) layers to produce spatially adapted attention weightings. Lastly, we also propose an optimized sheared‐grid sampling pattern which is designed to minimize contrast deviations between neighboring inversion recovery images and study its impact on reconstruction quality. We investigate the acquisition of cardiac T1 maps in diastasis with up to 1.14 × 1.14 mm^2^ resolution with similar scan time and acquisition window duration as conventional acquisitions. Additionally, the acceleration enables MOLLI acquisitions within a shortened acquisition window during the systole, which we also explored. We evaluated our method using both retrospectively undersampled (1865 patient scans) and prospectively acquired (27 volunteer scans) high‐resolution MOLLI data at 1.5 and 3 T. Reconstruction performance was assessed using common quantitative metrics, while T1 agreement with the clinical standard was analyzed through bullseye plots and Bland–Altman analysis.

## 2 Theory

### 2.1 Background of Unrolled Neural Networks

Multicoil MRI data is acquired as a raw complex‐valued signal organized in *k*‐space $k\in C^{n_{\mathrm{inv}}\times c\times h\times w}$, with $n_{\mathrm{inv}}$ being the number of acquired inversion recovery images, c being the number of coils and *h* and *w* being the image matrix sizes, respectively. The *k*‐space and the corresponding complex‐valued image $x\in C^{n_{\mathrm{inv}}\times c\times h\times w}$ are coupled via the forwarding encoding operator:

(1) $$k_{i,j}=E_{i}x_{i,j}+\epsilon =M_{i}{FS}_{j}x_{i,j}+\epsilon ,$$

with M being the undersampling mask, F being the Fourier Transform, S the coil sensitivity maps, ϵ noise, i the inversion recovery index, j the coil index in the range of [0,c−1], and x being the coil images. Let E=MFS be the forward encoding operator and its Hermitian $E^{H}$ the adjoint operator. We formulate the following regularized inverse problem to obtain x from an undersampled *k*‐space:

(2) $$\hat{x}=\underset{x}{\text{argmin}}{\left|\left|\mathrm{Ex}-k\right|\right|}_{2}^{2}+R\left(x\right),$$

where R is a regularization term. We opt to solve this problem using a neural network resembling gradient descent iterations. Following the formulation introduced in Sriram et al. [39] we define the following update rule in *k*‐space applied within each cascade n:

(3) $$k^{\left[n+1\right]}=k^{\left[n\right]}-\lambda_{n}\left(k^{\left[n\right]}-k^{\left[0\right]}\right)-EG^{\left[n\right]}\left(E^{H}k^{\left[n\right]}\right),$$

where $\lambda_{n}$ is the n‐th entry of a vector of learnable weights and G is a neural network backbone for regularization in the image space. Equation (3) updates the *k*‐space using a weighted data consistency (DC) term as well as a learned regularization term. After the final cascade N−1 the reconstructed image is obtained by applying the adjoint operator: $x^{\left[N\right]}=E^{H}k^{\left[N\right]}$.

## 3 Methods

### 3.1 MappingVN and Acquisition Strategy

We propose a modified E2E VarNet [39], termed MappingVN [53] in the following, specifically designed to reconstruct an entire accelerated MOLLI image set. The network solves the problem formulated in Equation (2). Our modifications include an additional set of convolution operations across the contrast dimension as well as pSE layers. We further make use of a sheared‐grid sampling pattern during the acquisition. The following subsections provide a detailed overview of all modifications.

#### 3.1.1 Sheared‐Grid Sampling Pattern

**FIGURE 1:**
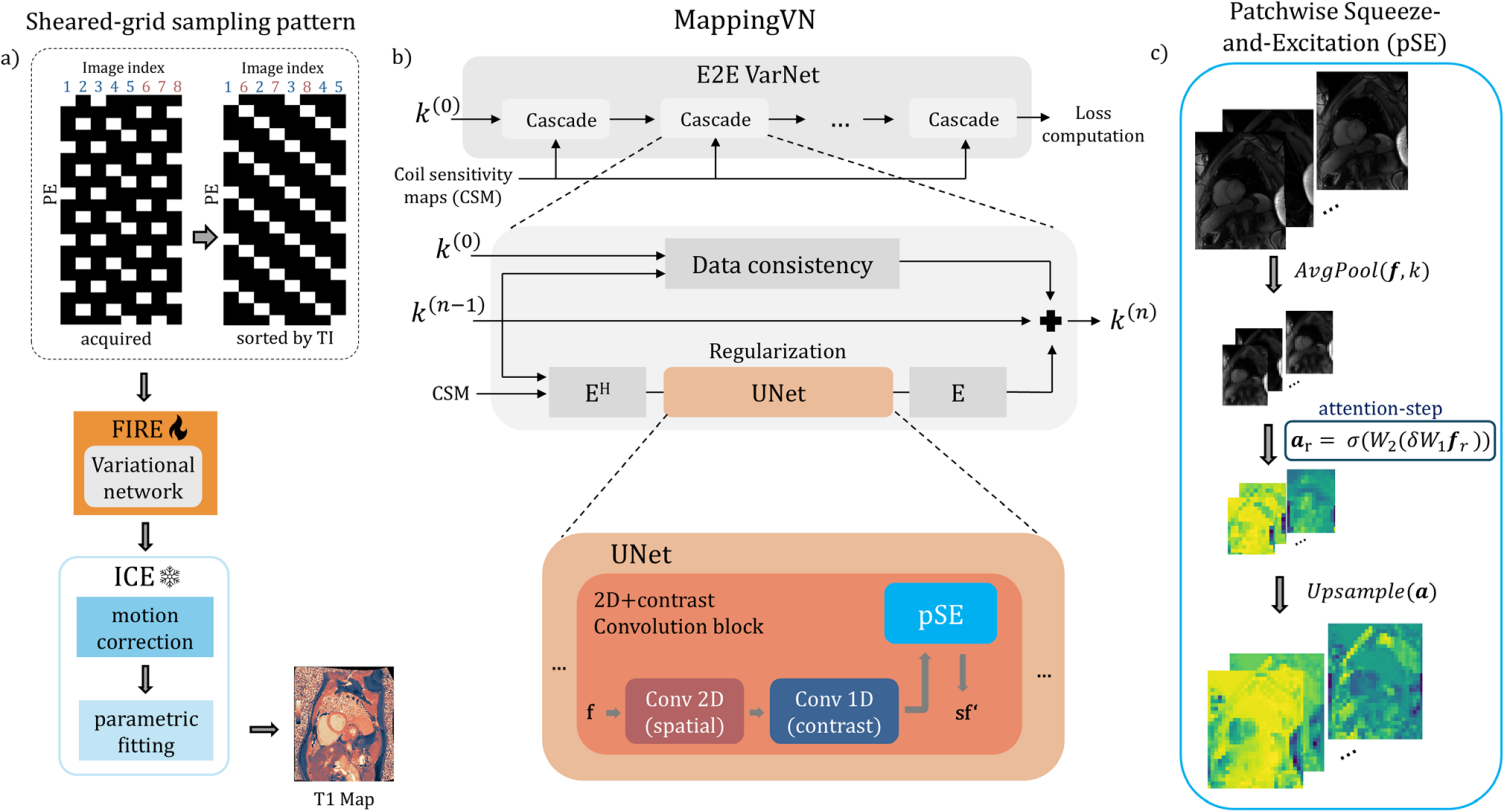
(a) Overview over the inline integration of the method. Using the FIRE prototype, our approach is integrated into an existing reconstruction pipeline for T1 mapping, implemented in a vendor reconstruction environment (ICE). (b) Visualization of the proposed MappingVN architecture. Colored modules symbolize modifications when compared to the standard VarNet. (c) Illustration of the pSE layer formulation. Following the calculation described in Section 3.1.3, full scale attention maps are computed from input feature maps within each 2D + contrast convolution block.

MOLLI inversion recovery images are typically acquired using an equispaced sampling pattern with *R* = 2. To further reduce acquisition time for individual shots, the auto calibration signal (ACS), composed of 36 *k*‐space lines and used for coil sensitivity and GRAPPA‐kernel estimation, is acquired only once at the end of the scan. Rather than solely increasing the acceleration rate to our target acceleration rate, we adapt the sampling strategy for our method. We replace the standard equispaced sampling pattern by a sheared‐grid pattern [54], that is shifted by a factor of $i_{\mathrm{inv}}\;\mathrm{mod}\;R$ in phase encoding direction, where $i_{\mathrm{inv}}$ refers to the image index in the image set. Previous work has shown, that this sampling strategy results in overlapping aliasing ghosts, whose structured nature facilitate more effective suppression during reconstruction [54, 55]. To perform the T1 map fitting step said images must be re‐ordered by TI, relative to the last preceding inversion pulse. After this re‐ordering, the inversion recovery samples roughly resemble the underlying exponential T1 decay, resulting in neighboring contrasts that deviate less severely from each other, compared to the unordered case. We therefore propose to use a sampling pattern that resembles a sheared‐grid pattern after the above‐mentioned re‐ordering was performed (see Figure 1a), leading to a network input with regularly overlapping aliasing ghosts and minimized contrast deviations across the inversion recovery images.

#### 3.1.2 2D ± Contrast Regularization

Following the E2E VarNet work, we employ U‐Nets for regularization in image space. The convolution blocks were modified to include 2‐D convolutions along the spatial dimensions and 1D convolutions along the contrast dimension (i.e., across inversion recovery images) to allow the network to make use of redundant anatomical information across different contrasts when performing regularization. Each convolution is followed by an InstanceNorm [56] and a LeakyReLU [57] activation function, forming the proposed convolution block. Two such convolution blocks are applied sequentially per U‐Net stage. In the encoder path of the U‐Net, average pooling is used to downsample input features while nearest‐neighbor interpolation is used for upsampling during the decoder path. The contrast dimension remains unaffected by any up‐ and downsampling operations. Architectural details are depicted in Figures 1b and S1.

#### 3.1.3 pSE Layers

In addition to 2D + contrast convolutions, we propose incorporating channel attention layers along the contrast dimension [58, 59], as illustrated in Figure 1. Let $f\in R^{b\times \mathrm{ch}\times n_{\mathrm{inv}}\times h^{\prime }\times w^{\prime }}$ be the feature map output of a convolution block at an arbitrary U‐Net stage, with b and ch being the batch size and channel size, respectively. First, f is padded to ensure divisibility by the chosen patch size p. The feature map is then divided into patches of size $b\times \mathrm{ch}\times n_{\mathrm{inv}}\times p\times p$ and Squeeze‐and‐Excitation computation [52] is applied:

$$a_{r}=\sigma \left(W_{2}\delta \left(W_{1}\mathrm{GAP}\left(f_{r}\right)\right)\right),$$

where σ represents the sigmoid function, δ represents the ReLU function [60], GAP denotes global average pooling, $W_{1}$ and $W_{2}$ are learnable weights of size $n_{\mathrm{inv}}\times n_{\mathrm{inv}}$ applied using matrix–vector multiplication, r is the patch index and $a_{r}$ is the final attention vector of size $b\times \mathrm{ch}\times n_{\mathrm{inv}}\times 1\times 1$ computed for patch r. The attention vectors are stacked to a low‐resolution attention map of size $b\times \mathrm{ch}\times n_{\mathrm{inv}}\times h/p\times w/p$ and finally upscaled using nearest‐neighbor interpolation to produce a full‐sized attention map. This map is multiplied with corresponding feature maps to activate the most significant regularization contributions. By using pSE layers the network gains the ability to re‐weight and focus regularization updates, which can be of help to extract the most meaningful contrast variations.

#### 3.1.4 Inline MR Scanner Integration

The proposed method was integrated inline into an existing pipeline for cardiac T1 Mapping using the framework for image reconstruction environments (FIRE) prototype [61]. The MappingVN replaces GRAPPA for image reconstruction, while the steps for motion correction [62] and parametric fitting [63] remain unchanged, utilizing standard vendor algorithms. This modified pipeline was used in all experiments, allowing direct comparisons of resulting T1 maps with those from the unmodified vendor pipeline. The proposed sheared‐grid sampling pattern was implemented as a prototype modification of the vendor MOLLI sequence for prospective studies.

#### 3.1.5 Choice of Target Acceleration Rate

To achieve a spatial resolution of 1.14 × 1.14 mm^2^ on 3T and 1.25 × 1.25 mm^2^ on 1.5T within a sufficiently short acquisition window, an acceleration factor of R=4 was found to be appropriate. Therefore, said acceleration rate was used for all experiments performed. Nevertheless, we did perform additional experiments with higher acceleration rates to also investigate the robustness of our method.

#### 3.1.6 Network Training and Implementation

Figure 1 shows the MappingVN architecture, comprising 15 cascades of shallow 2D + contrast U‐Nets. Initial experiments found this configuration optimal for our application and it is in line with literature [64]. pSE layers are applied after every second consecutive convolution block within each U‐Net stage, using a patch size of p=8. Following Vornehm et al. [64], phase cycling is used within each cascade as data augmentation to ensure correct phase output by preventing shortcut learning. A random factor q∈[0,2π] is sampled to rotate the input and output phases of the regularization network by q and −q, respectively. The inputs to the MappingVN consist of $n_{\mathrm{inv}}=8$ inversion recovery images from a retrospectively undersampled MOLLI acquisition, corresponding undersampling masks and coil sensitivity maps, computed using ESPiRIT [65]. Images were sorted by increasing TI prior to MappingVN processing. Supervised training was performed on retrospectively undersampled data to the reference data using a structural similarity index measure (SSIM) loss function. Training was conducted on 2 Nvidia RTX A100 GPUs using an Adam optimizer [66] with a learning rate of 0.005, multiplied by a factor of γ=0.1 every 40 epochs, and a batch size of 1.

### 3.2 Data

#### 3.2.1 Retrospective Data

A dataset consisting of 1750 MOLLI 5(3)3 scans in 1750 consecutive patients (626 female) referred to a cardiovascular magnetic resonance (CMR) exam was used for training, validation and retrospective testing (Retrospective‐3T). All scans were acquired on clinical 3T systems (MAGNETOM Skyra, Vida, Lumina, Cima.X, Siemens Healthineers, Forchheim, Germany) in mid‐ventricular short axis orientation using a vendor MOLLI T1 mapping sequence. Scans were performed during a breath hold using ECG‐triggering. The scan duration and acquisition window varied depending on the subject's heart rate.

A second test set (Retrospective‐1.5T) included 115 MOLLI 5(3)3 scans in 115 consecutive patients (46 female) referred to a CMR exam acquired on clinical 1.5T systems (MAGNETOM Sola, Aera, Siemens Healthineers, Forchheim, Germany).

Both datasets used the same breathing instructions, ECG synchronization, and sequence protocols, with written informed consent obtained from all patients. Unlike the Retrospective‐3T dataset, the Retrospective‐1.5T dataset featured smaller FOVs, higher spatial resolution, and longer acquisition windows. Table 1 summarizes the main scan parameters.

**TABLE 1:** Overview of the most important scan parameters used to acquire the Retrospective‐1.5T and Retrospective‐3T datasets. All data were originally acquired using an acceleration rate of *R* = 2.

Parameter	Retrospective‐1.5T	Retrospective‐3T
TR (ms)	3.02 ± 0.012	2.53 ± 0.033
TE (ms)	1.26 ± 0.005	1.05 ± 0.021
Acquisition window (ms)	330 ± 16.6	160 ± 16.7
Flip angle (°)	35 ± 0	35 ± 0
Slice thickness (mm)	8 ± 0	8 ± 0
FOV (mm^2^)	282 ± 0 × 275 ± 13	356 ± 21 × 202 ± 20
Matrix size	256 ± 0 × 250 ± 12	252 ± 15 × 144 ± 14
Partial Fourier factor	7/8	7/8
Resolution (mm^2^)		
Acquired	1.10 × 1.66	1.41 × 2.13
Reconstructed	1.10 × 1.10	1.41 × 1.41

Abbreviations: FOV, field of view; TE, echo time; TR, repetition time.

Both datasets were acquired using *R* = 2. Corresponding GRAPPA‐interpolated *k*‐space data were regarded as fully‐sampled reference and retrospectively undersampled using the proposed sheared‐grid sampling pattern. The Retrospective‐3T dataset was split into 1600 scans for training, 50 for validation, and 100 for testing, while the Retrospective‐1.5T dataset was exclusively used for testing.

#### 3.2.2 Prospective Data (diastolic)

All volunteers gave written informed consent prior to data acquisition. For prospective testing, 17 MOLLI 5(3)3 scans (Prospective‐3T and Prospective‐1.5T) were acquired in volunteers without known cardiovascular disease on 1.5T (*n* = 7, 3 female) and 3T (*n* = 10, 2 female) clinical systems (MAGNETOM Sola, Vida, Lumina, Cima.X, Siemens Healthineers, Forchheim, Germany). Each subject underwent two scans: one using the unmodified vendor MOLLI sequence and another using the proposed research sequence prototype with a sheared‐grid sampling pattern, *R* = 4 and a resolution of 1.14 × 1.14 mm^2^ on 3T and 1.25 × 1.25 mm^2^ on 1.5T. Acquired data was reconstructed using GRAPPA‐2 (for scan 1), GRAPPA‐4 (for scan 2) and the MappingVN (for scan 2). An overview of relevant scan parameters can be found in Table 2a.

**TABLE 2:** Overview of the most important scan parameters used to acquire all prospective datasets both in diastole (a) and systole (b). Note: (a) Scan parameters used for the Prospective‐1.5T and Prospective‐3T datasets. Standard resolution data was acquired using an acceleration factor of *R* = 2 and an equispaced undersampling pattern while the high‐resolution data was acquired using an acceleration factor of *R* = 4 and the sheared grid pattern introduced in Section 3.1.1. (b) Scan parameters used for all systolic T1 maps. Per volunteer, three scans with different protocol settings and reconstruction methods were performed.

	(a) Acquired during diastole				(b) Acquired during systole		
Parameter	Prospective‐1.5T (high resolution)	Prospective‐3T (high resolution)	Prospective‐1.5T (standard resolution)	Prospective‐3T (standard resolution)	Scan 1	Scan 2	Scan 3
TR (ms)	2.86 ± 0.0	3.00 ± 0.067	2.66 ± 0.015	2.70 ± 0.036	2.56 ± 0.104	2.55 ± 0.099	2.55 ± 0.099
TE (ms)	1.19 ± 0	1.28 ± 0.030	1.10 ± 0.007	1.13 ± 0.017	1.07 ± 0.045	1.07 ± 0.042	1.07 ± 0.042
Acquisition window (ms)	157 ± 14.6	186 ± 28.1	161 ± 15.1	174 ± 28.8	173 ± 21.1	86 ± 9.9	86 ± 9.6
Flip angle (°)	35 ± 0	35 ± 0	35 ± 0	35 ± 0	35 ± 0	35 ± 0	35 ± 0
Slice thickness (mm)	8 ± 0	8 ± 0	8 ± 0	8 ± 0	8 ± 0	8 ± 0	8 ± 0
FOV (mm^2^)	360 ± 0 × 313 ± 29	364 ± 0 × 322 ± 46	360 ± 8 × 206 ± 19	360 ± 0 × 208 ± 34	360 ± 0 × 216 ± 24	360 ± 0 × 217 ± 22	360 ± 0 × 217 ± 22
Matrix size	288 ± 0 × 251 ± 23	320 ± 0 × 283 ± 41	242 ± 5 × 138 ± 13	256 ± 0 × 147 ± 24	256 ± 0 × 153 ± 17	256 ± 0 × 154 ± 16	256 ± 0 × 154 ± 16
Resolution (mm^2^)							
Acquired	1.25 × 1.25	1.14 × 1.14	1.49 × 2.27	1.41 × 2.13	1.41 × 2.13	1.41 × 2.13	1.41 × 2.13
Reconstructed	1.25 × 1.25	1.14 × 1.14	1.49 × 1.49	1.41 × 1.41	1.41 × 1.41	1.41 × 1.41	1.41 × 1.41
Acceleration	4	4	2	2	2	4	4
Partial Fourier factor	7/8	7/8	7/8	7/8	7/8	7/8	7/8
Reconstruction method					GRAPPA	GRAPPA	MappingVN
Sampling pattern	Sheared‐grid	Sheared‐grid	Equispaced	Equispaced	Equispaced	Equispaced	Sheared‐grid

Next to volunteers, two patients with diagnosed cardiovascular disease (patient 1: hypertrophic cardiomyopathy; patient 2: cardiac amyloidosis) were scanned for cardiac examination, including standard resolution and high‐resolution T1 mapping (for protocol settings, see Table 2a).

#### 3.2.3 Prospective Data (systolic)

Additionally, 10 MOLLI 5(3)3 volunteer scans were acquired during the systole on 3T (*n* = 9, 1 female) and 1.5T (*n* = 1, 0 female) systems. Each volunteer underwent three measurements: scan 1 with the unmodified vendor mapping protocol (mean acquisition window: 174 ms), scan 2 with the same protocol but *R* = 4 (mean acquisition window: 86 ms) and scan 3 using a sheared‐grid sampling pattern and *R* = 4 (mean acquisition window: 86 ms). All scans were acquired at a resolution of 1.41 × 2.13 mm^2^. Similar to the other prospective datasets, the acquired scans were reconstructed using GRAPPA‐2 (scan 1), GRAPPA‐4 (scan 2), and the MappingVN (scan 3). Before each scan set, a cine acquisition in the same orientation as the T1 maps determined the systolic phase relative to the ECG signal. An overview of relevant scan parameters is shown in Table 2b.

### 3.3 Evaluation

Our proposed method was evaluated for both reconstruction performance and T1 accuracy. An ablation study was conducted to assess the impact of each modification relative to the original E2E VarNet. Variants included: an unmodified VarNet, a VarNet with 2D + contrast convolutions, a VarNet with 2D + contrast convolutions and the sheared‐grid sampling pattern (MappingVN w/o pSE), and the MappingVN (all modifications). All networks were trained using the same data and training settings as described in Section 3.1.3. All reconstructions were compared to GRAPPA‐4.

#### 3.3.1 Reconstruction Performance

Reconstruction methods were evaluated using the retrospectively undersampled test datasets (Retrospective‐3T and Retrospective‐1.5T). Metrics included SSIM, peak signal‐to‐noise ratio (PSNR), and normalized mean squared error (NMSE), comparing reconstructed single‐shot images to GRAPPA‐2 references. Additionally, statistical significance was assessed using paired Student's *t*‐tests (α=0.05 with Bonferroni correction [67]) to compare all methods.

#### 3.3.2 T1 Mapping in diastasis (Retrospective Data)

For selected methods, myocardial T1 values were additionally investigated. To calculate T1 maps from reconstructed inversion recovery images, vendor‐implemented motion correction and parametric fitting steps were applied. Myocardium segmentation was performed using a nn‐U‐Net that was trained in‐house with clinical data, annotated by medical experts [68]. Segmentation was only performed on reference data to avoid potential errors caused by low SNR input. Using these segmentations, the mean T1 error in the myocardium between predicted and reference T1 maps was visualized using Bland–Altman plots. Corresponding data was also analyzed using Peason correlation and regression analysis. Additionally, the six mid‐ventricular AHA segments [69] were identified, and corresponding mean T1 values were reported as bullseye plots. As a measure of T1 map quality, the mean standard deviation (SD) values across the myocardium were reported for all methods.

#### 3.3.3 T1 Mapping in diastasis (Prospective Data)

A similar analysis was conducted for the MappingVN and GRAPPA‐4 using accelerated, high‐resolution MOLLI scans prospectively acquired in volunteers. Standard resolution T1 maps served as a reference (Table 2). Again, a nn‐U‐Net [68] was used to segment the myocardium in all prospectively acquired T1 maps. Segmentation was only performed on reference and MappingVN reconstructions, since elevated noise levels were expected when using GRAPPA‐4. As these datasets contain multiple consecutive scans of the same volunteer, a manual alignment of the myocardium for each pair of scans was conducted. If this alignment was not possible due to motion or acquisition artifacts, the sample was rejected (*n* = 2 in total). Following this procedure, the mean T1 values in six mid‐ventricular AHA segments were retrieved and reported using Bland–Altman plots. Again, correlation and regression analysis were performed and SD values across the myocardium were computed.

#### 3.3.4 T1 Mapping in systole (Prospective Data)

Finally, to demonstrate the benefits of a shortened acquisition window, T1 maps acquired during the systole were qualitatively evaluated by two expert readers with 25 and 7 years of experience. A Likert scale was used to rate “Image sharpness” (1: blurry, 2: slightly blurry, 3: sharp), “Motion artifact level” (1: artifacts, 2: minor artifacts, 3: artifact‐free), and “Noise level” (1: noisy, 2: slightly noisy, 3: noise‐free). Scores were analyzed for statistical significance using the Mann–Whitney U test.

## 4 Results

### 4.1 Reconstruction Performance

**FIGURE 2:**
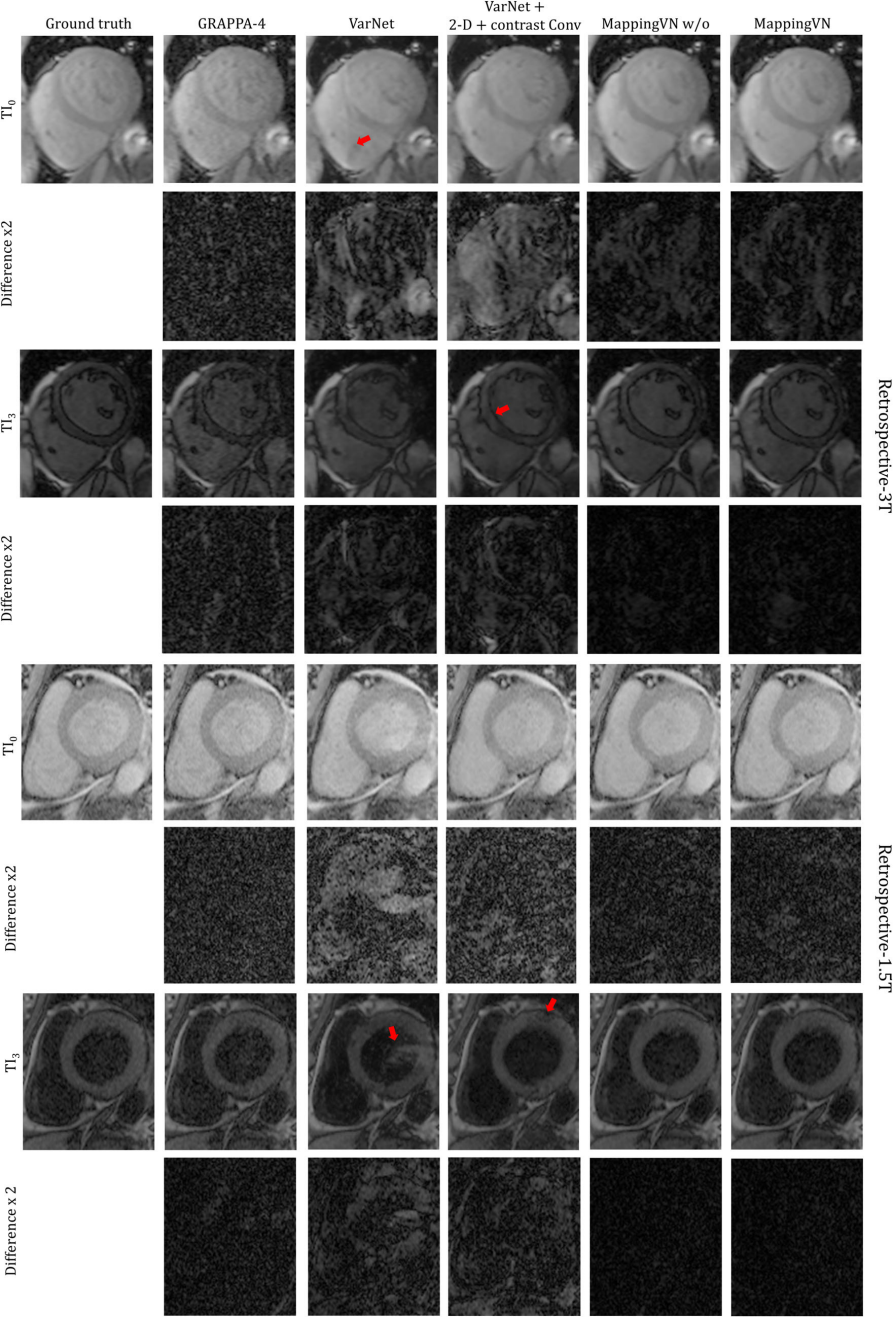
Example inversion recovery images reconstructed using the comparative methods (GRAPPA‐4, VarNet) and the ablations of the proposed MappingVN. Corresponding difference images compared to the ground truth are displayed below each image. Data from the Retrospective‐3T and Retrospective‐1.5T test sets were used. For each patient, the first and fourth image of the re‐ordered MOLLI images are shown. Percentile normalization was applied to improve the visibility, especially in the low signal images.

Table 3 indicates that at both tested field strengths and for our target acceleration rate *R* = 4, the MappingVN with and without pSE layers achieve the highest SSIM, PSNR, and the lowest NMSE scores. The lowest scores are reported for the unmodified VarNet. For SSIM values, only the difference between the MappingVN with and without pSE layers on 1.5 T data was insignificant (*p* = 1). For PSNR, all differences but the ones between GRAPPA‐4 and both MappingVN variants on 1.5 T data are significant (*p* < 3 × 10^−5^). Notably, these three methods were also the only ones that did not show significant differences in NMSE scores. Example reconstructions of inversion recovery images can be seen in Figure 2. Both MappingVN variants show only minimal deviations from the reference images. The GRAPPA‐4 reconstructions show increased noise while noticeable artifacts are visible for the unmodified VarNet and the VarNet with 2D + contrast convolutions. Table S1 shows a decrease in reconstruction performance for increased acceleration rates, which is also observable in the example reconstructions provided in Figure S2. The measured reconstruction time per Image set was 0.26 ± 0.13 s for the unmodified VarNet, 0.36 ± 0.19 s for the VarNet with 2‐D + contrast convolutions and the MappingVN without pSE layers, and 0.39 ± 0.21 s for the MappingVN. On average, motion correction took 9 s and parametric fitting took 3 s per Image set.

**TABLE 3:** Mean quantitative reconstruction metrics and corresponding standard deviations over the test cohorts achieved by GRAPPA‐4, the unmodified VarNet, the VarNet with 2D + contrast convolutions and the MappingVN with and without pSE layers, respectively. The SSIM, PSNR, and NMSE were computed using test data from the Retrospective‐1.5T and Retrospective‐3T datasets.

	GRAPPA‐4	Unmodified VarNet	VarNet (2D + contrast convolutions)	MappingVN (w/o pSE)	MappingVN
SSIM±3T	0.962 ± 0.015	0.920 ± 0.043	0.933 ± 0.034	0.982 ± 0.010	0.984 ± 0.008
SSIM±1.5T	0.935 ± 0.022	0.920 ± 0.028	0.882 ± 0.038	0.952 ± 0.017	0.952 ± 0.017
PSNR±3T	41.68 ± 2.43	34.48 ± 2.84	35.05 ± 2.519	42.44 ± 2.48	43.27 ± 2.40
PSNR±1.5T	40.09 ± 3.39	34.83 ± 2.67	34.25 ± 3.104	40.91 ± 2.66	40.88 ± 2.45
NMSE±3T	0.003 ± 0.001	0.018 ± 0.013	0.015 ± 0.009	0.003 ± 0.004	0.003 ± 0.002
NMSE±1.5T	0.006 ± 0.004	0.021 ± 0.011	0.024 ± 0.013	0.006 ± 0.002	0.006 ± 0.002

### 4.2 T1 Evaluation (Retrospective Data)

**FIGURE 3:**
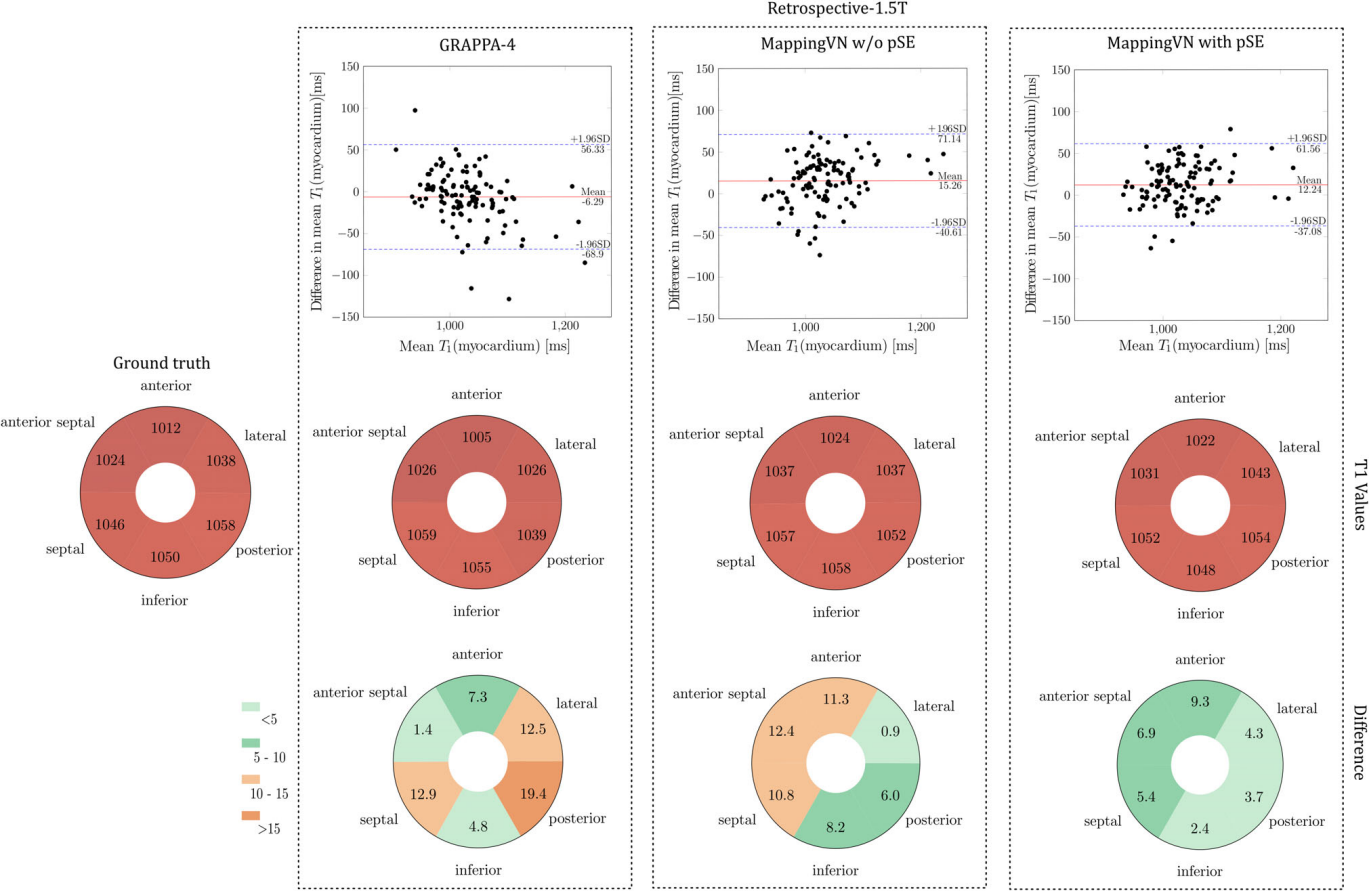
Results for the retrospective evaluation in diastole of T1 agreement using GRAPPA‐4 and the MappingVN with (proposed) and without (ablation) pSE layers for reconstruction. Data from the Retrospective‐1.5T dataset was used. In the Bland–Altman plots shown in the upper half of the figure, the red line symbolizes the mean T1 difference while corresponding limits of agreement are shown in dotted blue lines. Reference T1 values were subtracted from T1 values produced by the proposed method (proposed—reference). The bullseye plots shown in the first row of the lower half of the figure report mean T1 values in the six mid‐ventricular AHA segments for the ground truth and all evaluated methods. The second row shows bullseye plots with the T1 difference compared to the ground truth, color‐coded according to the legend shown on the left.

**FIGURE 4:**
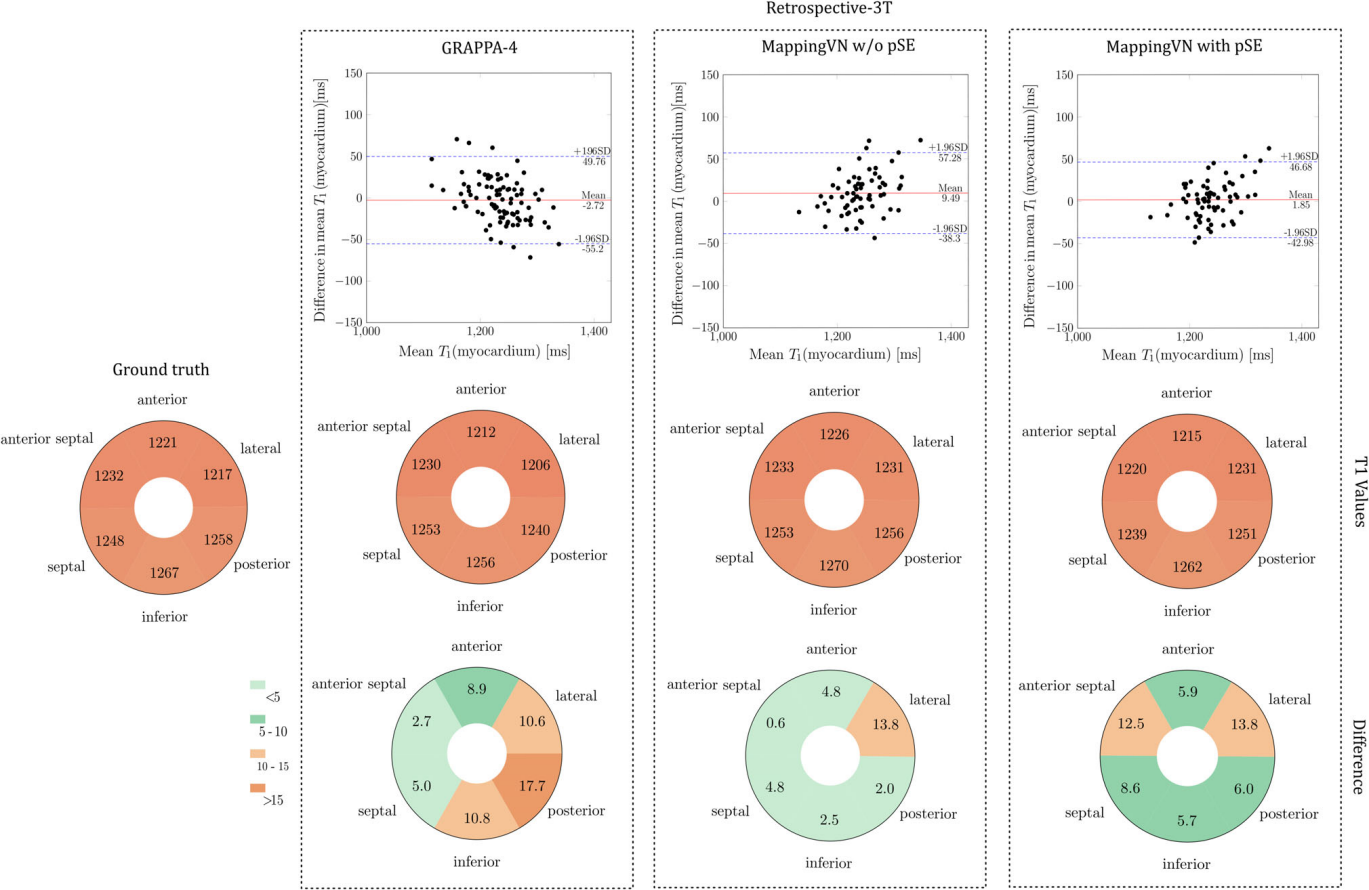
Results for the retrospective evaluation in diastole of T1 agreement using GRAPPA‐4 and the MappingVN with (proposed) and without (ablation) pSE layers for reconstruction. Data from the Retrospective‐3T test dataset was used. In the Bland–Altman plots shown in the upper half of the figure, the red line symbolizes the mean T1 difference while corresponding limits of agreement are shown in dotted blue lines. Reference T1 values were subtracted from T1 values produced by the proposed method (proposed—reference). The bullseye plots shown in the first row of the lower half of the figure report mean T1 values in the six mid‐ventricular AHA segments for the ground truth and all evaluated methods. The second row shows bullseye plots with the T1 difference compared to the ground truth, color‐coded according to the legend shown on the left.

The T1 accuracy of the MappingVN (with and without pSE) and GRAPPA‐4 was evaluated, with results presented in Bland–Altman plots and bullseye plots (Figures 3 and 4). Both MappingVN variants demonstrated low mean T1 differences (1.5T: pSE: 12.2 ms, without pSE: 15.3 ms; 3T: pSE: 1.9 ms, without pSE: 9.5 ms). However, MappingVN with pSE layers exhibited narrower limits of agreement (LoA) compared to MappingVN without pSE layers, particularly for 1.5T (LoA pSE: −37.1 to 61.6 ms; LoA without pSE: −40.6 to 71.1 ms) but also 3T data (LoA pSE: −43.0 to 46.7 ms; LoA without pSE: −38.3 to 57.3 ms). While GRAPPA‐4 showed low mean T1 differences, corresponding LoA were noticeably broader compared to the deep‐learning‐based methods. An analysis of mean T1 deviations across AHA segments revealed no notable segment‐specific differences for any of the methods investigated. Furthermore, correlation coefficients were above 0.8 for all methods, while all reported slopes were found to be between 0.98 and 1.1, see Figure S3. T1 map SD values across the myocardium were below 120 ms on 1.5T and below 75 ms on 3T for the MappingVN variants and the reference and above 140 ms on 1.5T and 105 ms on 3T for GRAPPA‐4, see Table S2a.

### 4.3 T1 Evaluation (Prospective Data)

**FIGURE 5:**
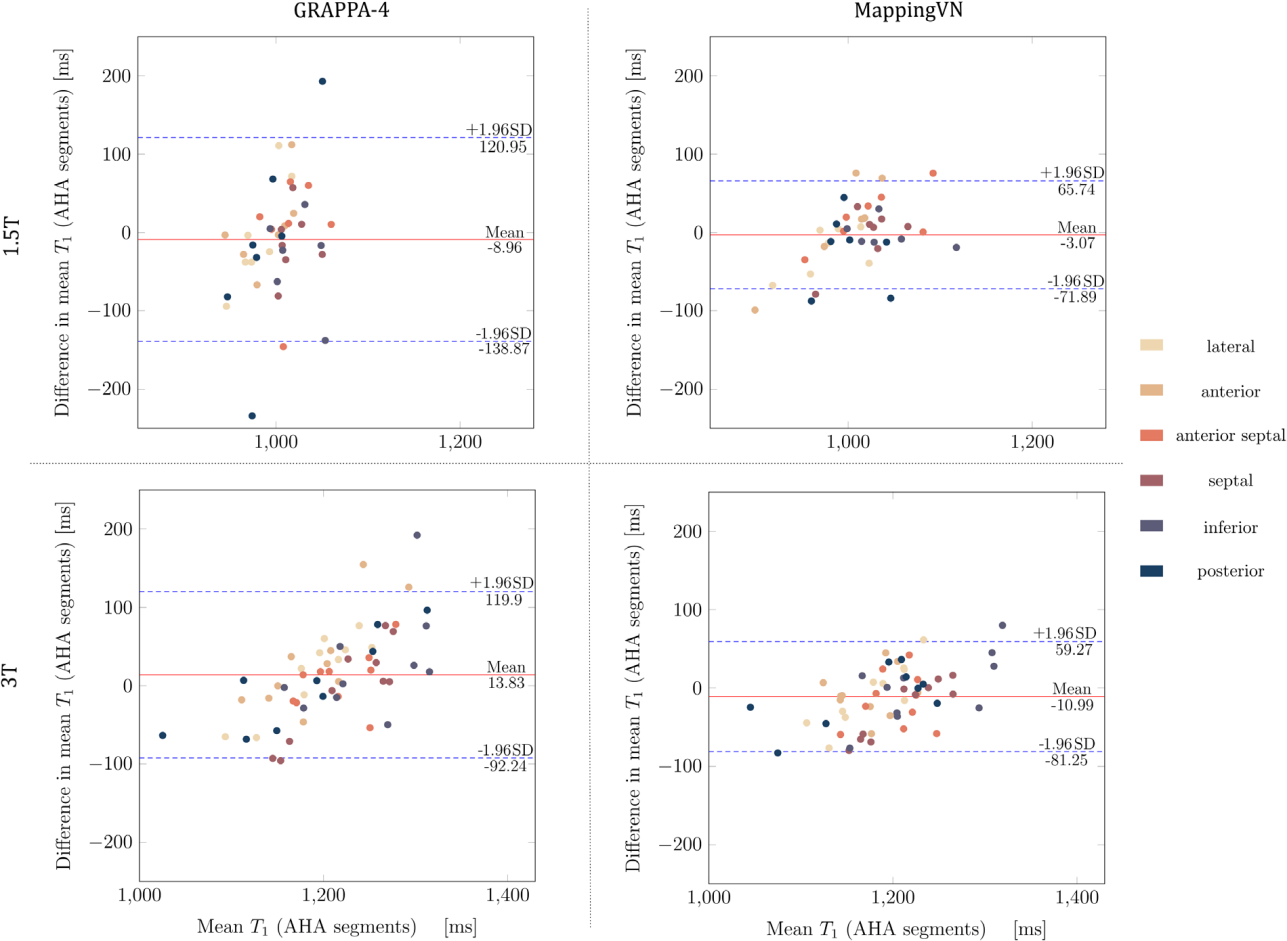
Result in prospective data in diastole for T1 agreement. Bland–Altman plots show the results for the evaluation of T1 agreement using GRAPPA‐4 and the MappingVN in prospectively acquired high‐resolution MOLLI acquisitions. Resulting T1 maps were compared to reference T1 maps in standard resolution, acquired in the same volunteer but in a different scan. Individual AHA segments rather than the mean T1 values across the whole myocardium were compared. Reference T1 values were subtracted from T1 values produced by the proposed method (proposed—reference).

**FIGURE 6:**
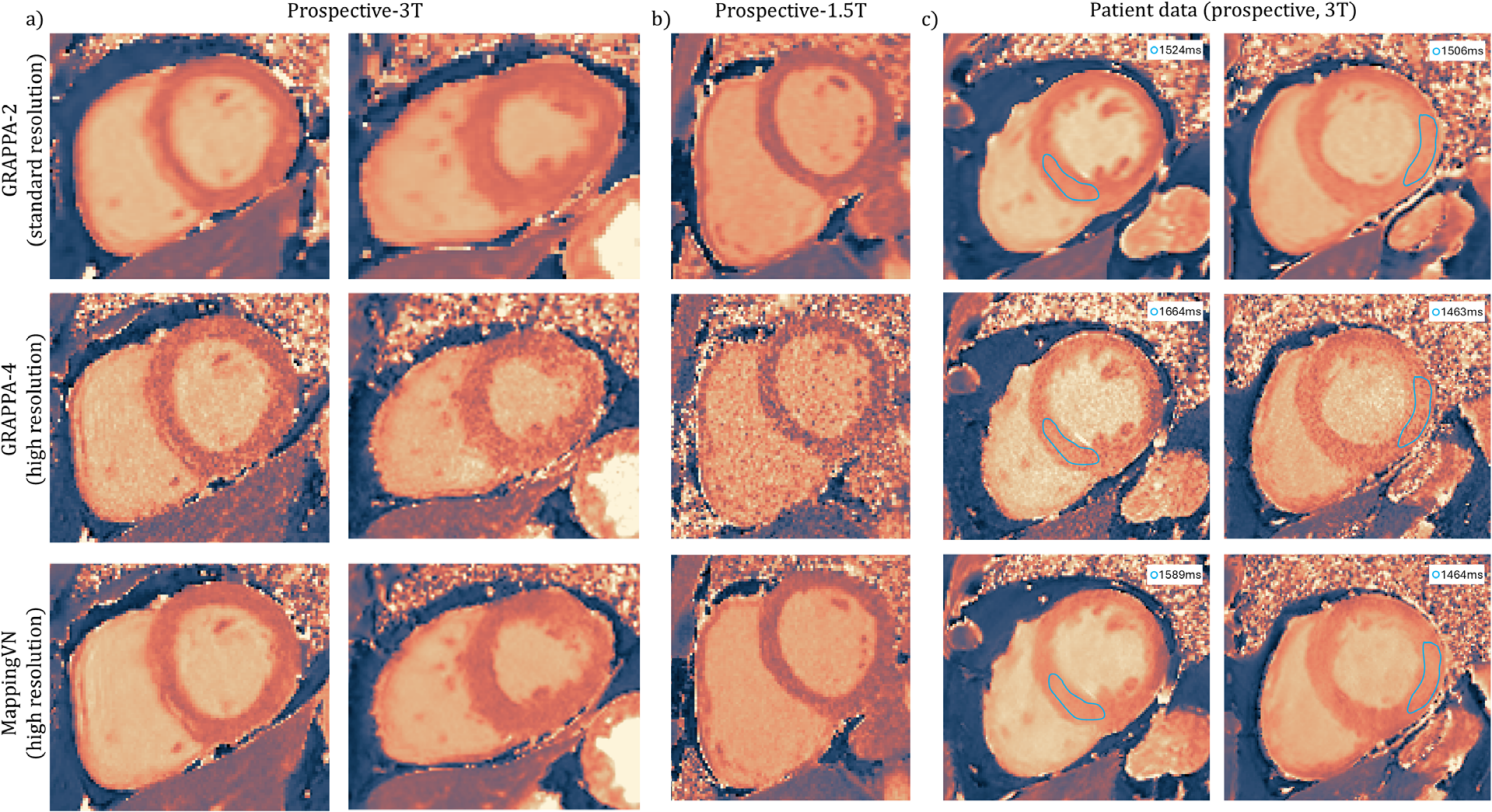
Example T1 maps acquired prospectively in diastole on both 1.5 and 3 T systems. Each column corresponds to data acquired for one volunteer (a and b) or patient (c). T1 maps acquired in standard resolution and reconstructed using GRAPPA‐2 as well as high‐resolution T1 maps reconstructed using GRAPPA‐4 and the proposed MappingVN are shown. Both patients were diagnosed with a form of cardiovascular disease and therefore show heavily impaired T1 values in the myocardium.

Figures 5 and S4 show the T1 agreement across the six mid‐ventricular AHA segments for T1 maps reconstructed using the MappingVN as well as GRAPPA‐4 and reference T1 maps. For both investigated field strengths, the mean T1 differences across AHA segments are below 14 ms for both methods. For the MappingVN, LoA range from −81.3 to 59.3 ms for 3T and −72.0 to 65.7 ms for 1.5T, while 78% of all analyzed segments show deviations below 50 ms. In contrast, GRAPPA‐4 exhibited noticeably broader LoA, ranging from −92.2 to 119.9 ms for 3T and −138.9 to 121.0 ms for 1.5T. Example T1 maps reconstructed using the MappingVN, GRAPPA‐4, and GRAPPA‐2 are shown in Figure 6. Pearson coefficients reported for the MappingVN were above 0.6 while GRAPPA‐4 showed values of 0.74 for 3T and −0.24 for 1.5T, see Figure S5. The T1 map SD values were 59.55 ms on 1.5T and 54.77 ms on 3T for the reference, 146.13 ms on 1.5T and 162.46 ms on 3T for GRAPPA‐4 and 72.83 ms on 1.5T and 83.83 ms on 3T for the MappingVN, see Table S2b.

### 4.4 T1 Evaluation in systole (Prospective Data)

**FIGURE 7:**
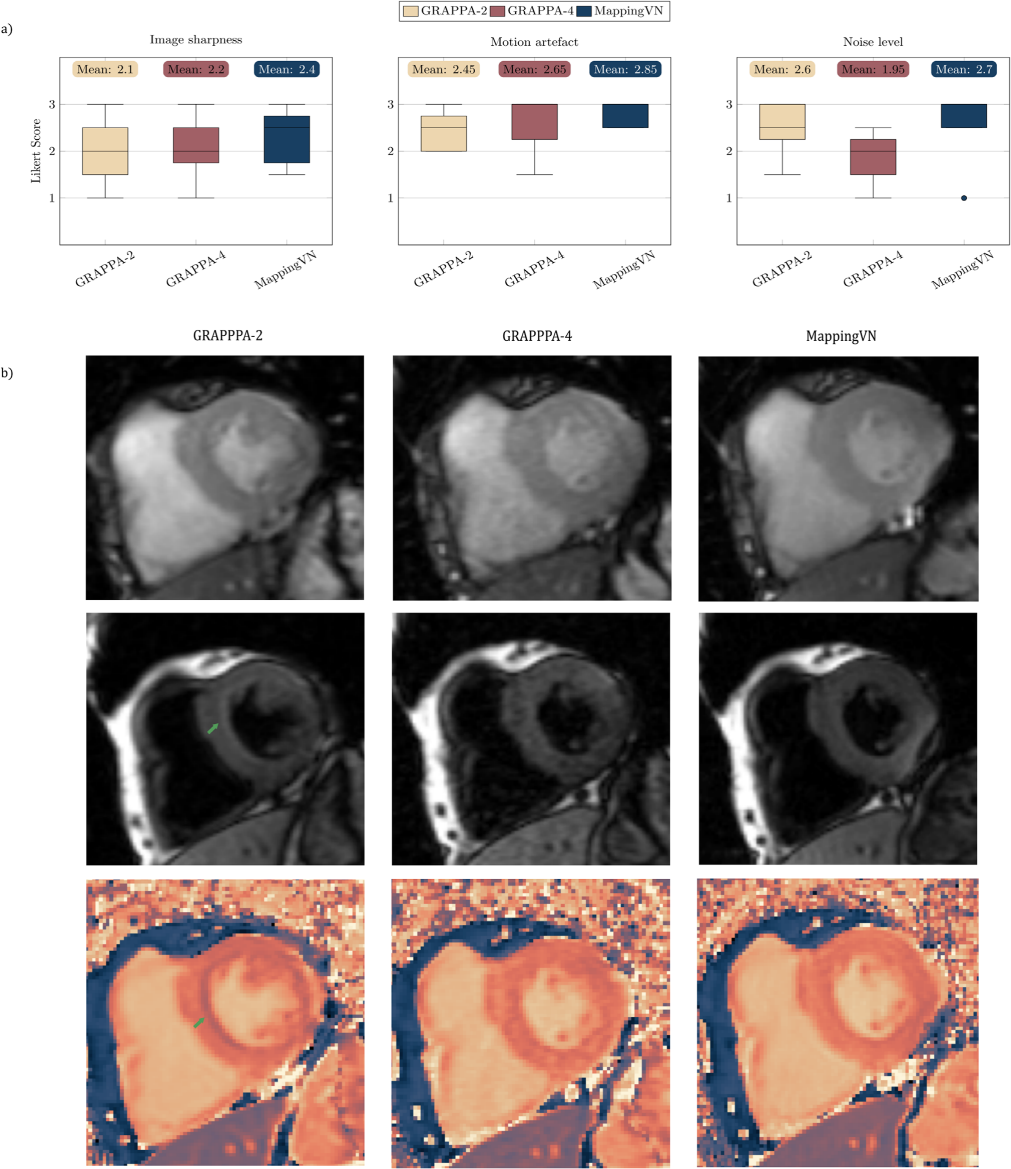
(a) Results of the reader study for the systolic T1 mapping experiment. The distribution of the scores is illustrated as boxplots. The scores of both readers were averaged. The individual ratings can be found in Figure S6. Corresponding mean scores are shown in color‐coded boxes. (b) Example inversion recovery images and corresponding T1 maps for MOLLI acquisitions in the systolic phase. The illustrated cases were reconstructed using GRAPPA‐2, GRAPPA‐4 and the proposed MappingVN, respectively. All data were acquired with the same spatial resolution but differing acquisition windows. The artifact visible in the MappingVN reconstruction of the inversion recovery image was found to be an acquisition artifact.

To assess the impact of a prolonged acquisition window, we evaluated our method for T1 mapping during systole. The results for the reader study, scoring reconstructed inversion recovery images and respective T1 maps in multiple categories, can be found in Figure 7a. T1 maps and image sets reconstructed using our proposed method achieved the best scores in all categories. Image sharpness for GRAPPA‐2 and GRAPPA‐4 was rated similarly; however, GRAPPA‐2 reconstructions exhibited more motion artifacts, and GRAPPA‐4 reconstructions were the noisiest. Furthermore, the difference in observed motion artifacts between GRAPPA‐2 and the MappingVN and the difference in noise level between GRAPPA‐4 and the MappingVN were found to be statistically significant (*p*‐values: 0.035 and 0.004). Figure 7b shows a comparison of systolic T1 maps reconstructed using GRAPPA‐2, GRAPPA‐4 and the MappingVN.

## 5 Discussion

In this work, we propose a deep‐learning‐based image reconstruction method that is capable of reconstructing accelerated inversion recovery images to achieve high‐resolution cardiac T1 mapping on both 1.5 and 3T systems. We proposed a sheared‐grid sampling pattern to optimize the aliasing artifact distribution in the single‐shot images. The entire reconstruction was implemented inline on the MR scanner. Experiments were performed for scans in diastasis (up to 1.14 × 1.14 mm^2^ in 186.08 ms acquisition window) and systole (1.41 × 2.13 mm^2^ within 86.12 ms acquisition window).

Our studies with retrospective data show that using an unmodified VarNet results in unsatisfactory reconstructions of MOLLI acquisitions. Adding 2D + contrast convolutions slightly improves performance, but residual artifacts remain visible (Figure 2). Utilizing the proposed sheared‐grid pattern significantly improves the image quality of reconstructed single‐shots, which is reflected in the reported metric scores (Table 3) and example reconstructions (Figure 2). While differences in SSIM scores for 3T data were statistically significant, the performance improvement of MappingVN compared to MappingVN without pSE layers was minor. The combination of the sheared‐grid sampling pattern and 2D + contrast convolutions substantially improved reconstruction performance and was essential to surpass GRAPPA‐4 as the comparison method.

The observations mentioned above are consistent for both 3T and previously unseen 1.5T data, highlighting the generalization capabilities of the MappingVN. However, metric scores for 1.5T data are slightly lower, likely due to increased noise levels and the absence of 1.5T training data. Additionally, it is worth noting that the Retrospective‐1.5T data was acquired with higher spatial resolution and a smaller FOV compared to the Retrospective‐3T data, resulting in generally noisier reference reconstructions due to reduced SNR. We attribute the lower metric scores for the 1.5T data to protocol differences arising because these datasets were acquired at different sites.

Performance improvements with the MappingVN were also evident in the evaluation of T1 accuracy using the retrospective datasets. All evaluated methods demonstrated overall good T1 agreement with the reference method (Figures 3 and 4). Furthermore, all methods showed good correlation with reference T1 values and regression analysis found no proportional bias, see Figure S3. Nevertheless, both MappingVN variants slightly outperformed GRAPPA‐4, achieving narrower LoA and lower mean T1 deviations. The elevated T1 map SD values (Table S2) also indicate noisy T1 maps for GRAPPA‐4. When comparing the tested MappingVN variants to each other, a slight performance increase can be observed when using pSE layers. Especially for 1.5T data, the SD of mean T1 differences (Figures 3 and 4) is noticeably reduced for the network utilizing the attention mechanism. Although the impact of the pSE layers is not as significant as the combination of the other two modifications, the performed quantitative evaluation shows improved robustness and slightly better T1 agreement with reference data when utilizing this feature. Performance improvements when using the pSE layers were clearer in the aspect of T1 accuracy compared to metrics computed purely on inversion recovery images. Similar observations about classical reconstruction metrics not fully reflecting the performance of the downstream task were also reported in recent literature [36].

In this work, our goal was to establish the applicability of our method for high‐resolution cardiac T1 mapping and to assess its performance in a prospective setting. The Bland–Altman plots in Figure 5 visualize the feasibility of the MappingVN in terms of T1 accuracy when compared to conventional MOLLI acquisitions. The low mean T1 difference and narrow LoA for both 1.5 and 3T data imply good T1 agreement with the reference. Approximately 80% of analyzed AHA segments in the deep learning‐reconstructed data showed deviations under 50 ms, with no significant outliers exceeding 100 ms. Corresponding Pearson correlation was found to be strong, but the regression analysis showed a slight proportional bias, see Figure S5. The data reconstructed with our method was acquired in a different resolution and in separate scans. Hence, a certain degree of deviation can be expected, an aspect that has also been reported in previous studies [8, 9, 70]. Additionally, a slight systematic underestimation of T1 can be observed for the high‐resolution T1 maps. While the deviation may be caused by the MappingVN itself, it could also result from reduced partial volume effects in the myocardium due to increased resolution. A similar reduction occurs when acquiring T1 maps in systole. Corresponding literature reports similar observations with regards to underestimation of T1 with reduced partial volume [10, 11, 71]. Compared to GRAPPA‐4, the proposed MappingVN clearly outperforms the conventional method in terms of T1 precision. We assume the decreased SNR in the GRAPPA‐4 reconstructions, underlined by the drastically increased T1 map SD values reported in Table S2, to cause said T1 imprecisions. A qualitative comparison of conventional and high‐resolution T1 maps reconstructed using the MappingVN yields improved visibility of small structures, see Figure 6a–c. Papillary muscles, for example, appear visibly sharper, which on its own can be a diagnostically relevant factor [72]. In patients (Figure 6c), the same observations can be made. Abnormally high T1 values in the myocardium of both patients can be seen for the MappingVN reconstruction as well as for the clinical reference, which correlates with the corresponding diagnoses. The potential improvements for the depiction of small lesions could not be investigated, as no fitting patient data was available.

In this work, we not only investigated the acquisition of high‐resolution T1 maps in diastasis, but also studied potential benefits of applying our approach for T1 mapping during the systole. The performed reader study showed that shortening the acquisition window by increasing the acceleration rate does reduce the appearance of motion artifacts. A similar statement can be made in relation to observed image sharpness of single‐shot images and corresponding T1 maps. Both observations are reflected in the reader scores, rating MappingVN and GRAPPA‐4 reconstructions higher than conventional ones. Although GRAPPA‐4 produced sharp inversion recovery images with minimal motion artifacts, the reconstructions showed increased noise, a limitation not observed with the MappingVN. This indicates high suitability of the MappingVN for T1 mapping during systole. Inter‐reader agreement was low indicated by low Kappa values of under 0.6 for every category except the noise level of MappingVN reconstructions (see Figure S6). Nevertheless, we saw on average improved scores for the proposed approach over the comparative methods, see Figure S6 for the individual rater scores. It is important to note that previously published work on systolic T1 mapping uses decreased spatial resolution to counteract the downsides of acquiring MOLLI data with lower acceleration rates [10, 11, 71]. We knowingly chose to acquire all T1 maps with a standard resolution of 1.41 × 2.13 mm^2^ to better investigate the acquisition window duration's effects on the resulting image quality. Our choice of resolution was also used to acquire the Retrospective‐3T dataset and aligns with recommendations by the field [73, 74]. We further want to add that our chosen resolution setting is desirable for systolic T1 mapping as it allows for improved visibility of small structures and a better comparison of diastolic and systolic T1 maps. Proper acquisitions using said spatial resolution were only possible when using the MappingVN instead of GRAPPA for reconstruction, as previously discussed.

### 5.1 Limitations and Outlook

In the scope of this study, mainly an acceleration rate of *R* = 4 was investigated, as it was deemed sufficient to achieve a target resolution of 1.14 × 1.14 mm^2^ on 3T and 1.25 × 1.25 mm^2^ on 1.5T without heavily prolonging the acquisition window. Increasing the acceleration even more would shorten the acquisition window further but potentially also compromise the reconstruction quality, as partly shown in Table S1 and Figure S2. Although the results for *R* = 5 are promising, a clear decrease in performance is observable with even higher acceleration rates. Nevertheless, we do want to point out that our method was developed with an acceleration rate of *R* = 4 in mind, as it allows for a clinically meaningful increase in spatial resolution.

The acquisition of fully‐sampled MOLLI data with clinically acceptable resolution and without motion artifacts is unfeasible, given the constrained time window in diastasis. Hence, all retrospectively acquired data used *R* = 2 and GRAPPA‐2 for reconstruction, which might be a potential source of bias for the network in this supervised learning setting. Reference reconstructions were carefully checked for sufficient quality, and no apparent biases were observed. Although being challenging to acquire, higher resolution MOLLI training data could potentially be beneficial for network performance.

Although the MappingVN did perform well on unseen 1.5T data, we expect a performance increase with the addition of 1.5T data to the training, ideally with resolution settings similar to those in the Retrospective‐3T dataset. The observed generalizability capabilities could potentially enable high‐resolution postcontrast T1 mapping without re‐training the model on postcontrast data.

The evaluation results on prospectively acquired high‐resolution data underline the feasibility of our proposed approach. Nevertheless, it was prospectively tested for only one vendor in a single center. Future clinical evaluation is necessary to investigate the potential benefits of high‐resolution T1 mapping.

## 6 Conclusion

In this work we introduced a deep‐learning‐based image reconstruction method that enables the acquisition of high‐resolution MOLLI T1 maps. When utilizing the proposed MappingVN, an acquired and reconstructed spatial resolution of 1.25 × 1.25 mm^2^ on 1.5T and 1.14 × 1.14 mm^2^ on 3T can be achieved in diastasis without an increase in scan time or breath hold duration. In addition, we were able to acquire T1 maps in systole for an acquired spatial resolution of 1.41 × 2.13 mm^2^ (interpolated to 1.41 mm^2^ isotropic) in a shortened acquisition window of 86 ms on average. Our approach was evaluated for reconstruction quality of inversion recovery images and T1 agreement with reference measurements using both retrospectively and prospectively acquired data.

## Funding

The authors have nothing to report.

## Conflicts of Interest

Daniel Amsel receives PhD funding from Siemens Healthineers. Kelvin Chow is an employee of Siemens Healthcare Ltd. Daniel Giese, Michaela Schmid, and Jens Wetzl are employee of Siemens Healthineers.

## Supporting information


**Figure S1.** Detailed illustration of the U‐Net‐based regularizer (a) as well as the convolution block (b) and the transposed convolution block (c) utilized within the proposed U‐Net architecture.
**Figure S2.** Example inversion recovery images retrospectively undersampled with acceleration rates ranging from 4 to 7 and reconstructed with MappingVN networks trained for the respective acceleration rate. Data from the Retrospective‐3T and Retrospective‐1.5T test sets was used. For each patient, the first and forth image of the re‐ordered MOLLI images are shown. Percentile normalization was applied to improve the visibility, especially in the low signal images.
**Figure S3.** Regression analysis for the T1 comparisons performed using the Retrospective‐3T and Retrospective‐1.5T datasets. The plots show data points (black dots), the linear fit (red) and the corresponding 95% confidence intervals (gray) for the comparison of T1 maps reconstructed using GRAPPA‐4 and the MappingVN with and without pSE layers to reference T1 maps. The slope and intercept values as well as the Pearson coefficient r are given in the top left corner of each subplot.
**Figure S4.** Result in prospective data in diastole for T1 agreement. Bland–Altman plots show the results for the evaluation of T1 agreement using GRAPPA‐4 and the MappingVN in prospectively acquired high‐resolution MOLLI acquisitions. Resulting T1 maps were compared to reference T1 maps in standard resolution, acquired in the same volunteer but in a different scan. Reference T1 values were subtracted from T1 values produced by the proposed method (proposed—reference).
**Figure S5.** Regression analysis for the T1 comparisons performed using the Prospective‐3T and Prospective‐1.5T datasets. The plots show data points, the linear fit and the corresponding 95% confidence intervals for the comparison of high‐resolution T1 maps reconstructed using GRAPPA‐4 and the MappingVN to reference T1 maps in standard resolution. The slope and intercept values as well as the Pearson coefficient *r* are given in the top left corner of each subplot.
**Figure S6.** Results of the reader study for the systolic T1 mapping experiment, for (a) reader 1 and (b) reader 2. Corresponding mean scores are shown in color‐coded boxes. The table in subfigure (c) shows corresponding coefficients computed using quadratically weighted Cohens Kappa.
**Table S1.** Achieved metric scores when reconstructing retrospectively undersampled test data with acceleration rates between 4 and 7 using MappingVN networks trained for the respective acceleration rates.
**Table S2.** Mean standard deviation (SD) values across the myocardium of T1 maps for all retrospective (a) and prospective (b) experiments. The mean SD is reported for all methods evaluated in terms of T1 accuracy.

## Data Availability

Research data are not shared.
