# Supplementary material for: Deep‐Learning‐Based Image Reconstruction to Improve End‐Diastolic and Systolic Cardiac T1 Mapping
Source: Magn Reson Med. 2026 Mar 19;96(2):892–907. doi: 10.1002/mrm.70353 (PMC13269227; doi:10.1002/mrm.70353)
Supplement: Supplementary file 1 — Figure S1. Detailed illustration of the U‐Net‐based regularizer (a) as well as the convolution block (b) and the transposed convolution block (c) utilized within the proposed U‐Net architecture. Figure S2. Example inversion recovery images retrospectively undersampled with acceleration rates ranging from 4 to 7 and reconstructed with MappingVN networks trained for the respective acceleration rate. Data from the Retrospective‐3T and Retrospective‐1.5T test sets was used. For each patient, the first and forth image of the re‐ordered MOLLI images are shown. Percentile normalization was applied to improve the visibility, especially in the low signal images. Figure S3. Regression analysis for the T1 comparisons performed using the Retrospective‐3T and Retrospective‐1.5T datasets. The plots show data points (black dots), the linear fit (red) and the corresponding 95% confidence intervals (gray) for the comparison of T1 maps reconstructed using GRAPPA‐4 and the MappingVN with and without pSE layers to reference T1 maps. The slope and intercept values as well as the Pearson coefficient r are given in the top left corner of each subplot. Figure S4. Result in prospective data in diastole for T1 agreement. Bland–Altman plots show the results for the evaluation of T1 agreement using GRAPPA‐4 and the MappingVN in prospectively acquired high‐resolution MOLLI acquisitions. Resulting T1 maps were compared to reference T1 maps in standard resolution, acquired in the same volunteer but in a different scan. Reference T1 values were subtracted from T1 values produced by the proposed method (proposed—reference). Figure S5. Regression analysis for the T1 comparisons performed using the Prospective‐3T and Prospective‐1.5T datasets. The plots show data points, the linear fit and the corresponding 95% confidence intervals for the comparison of high‐resolution T1 maps reconstructed using GRAPPA‐4 and the MappingVN to reference T1 maps in standard resolution. The slope and intercept values as [file MRM-96-892-s001.docx]

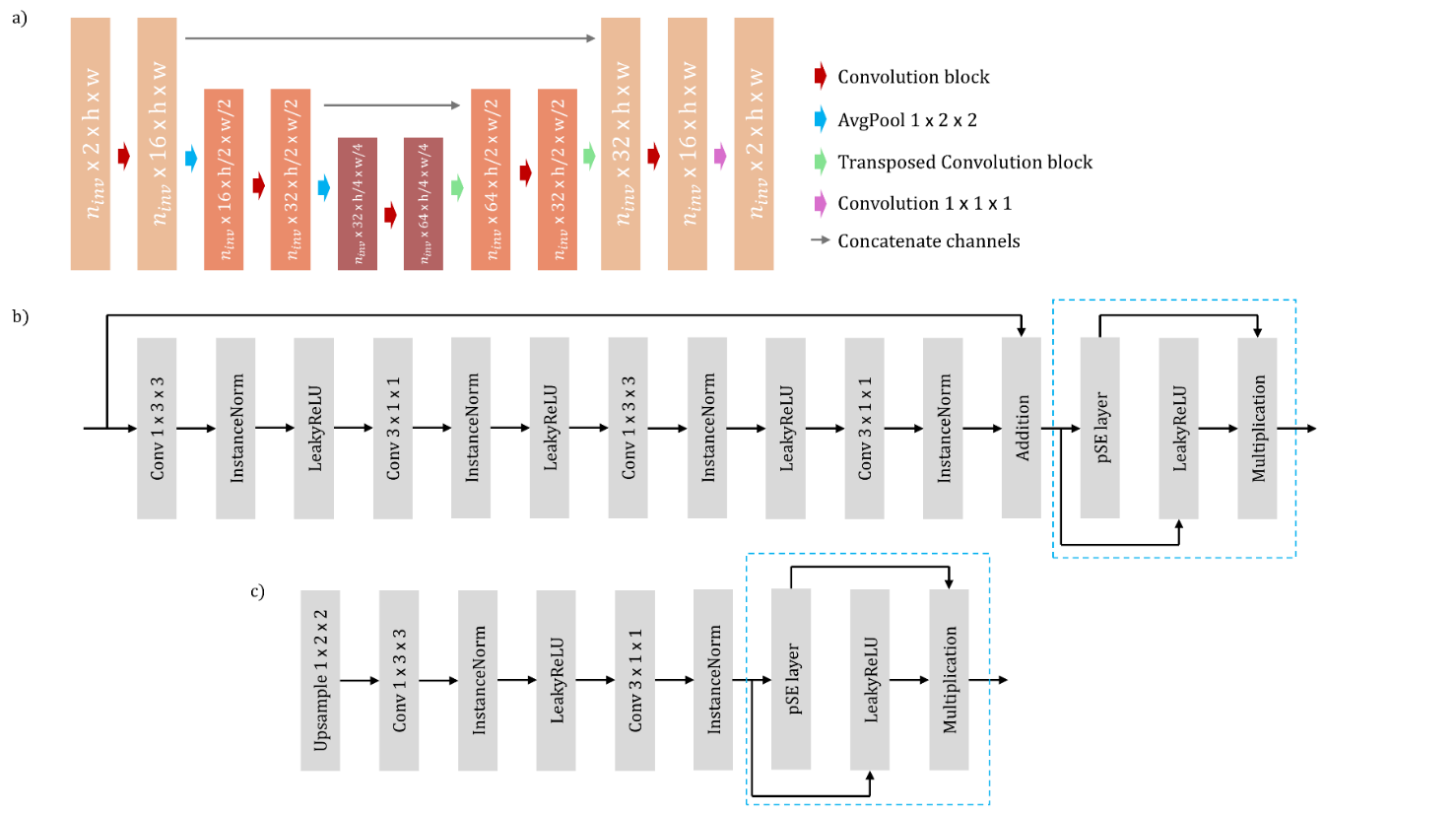


Supporting Information Figure S1: Detailed illustration of the U-Net-based regularizer (a) as well as the convolution block (b) and the transposed convolution block (c) utilized within the proposed U-Net architecture.


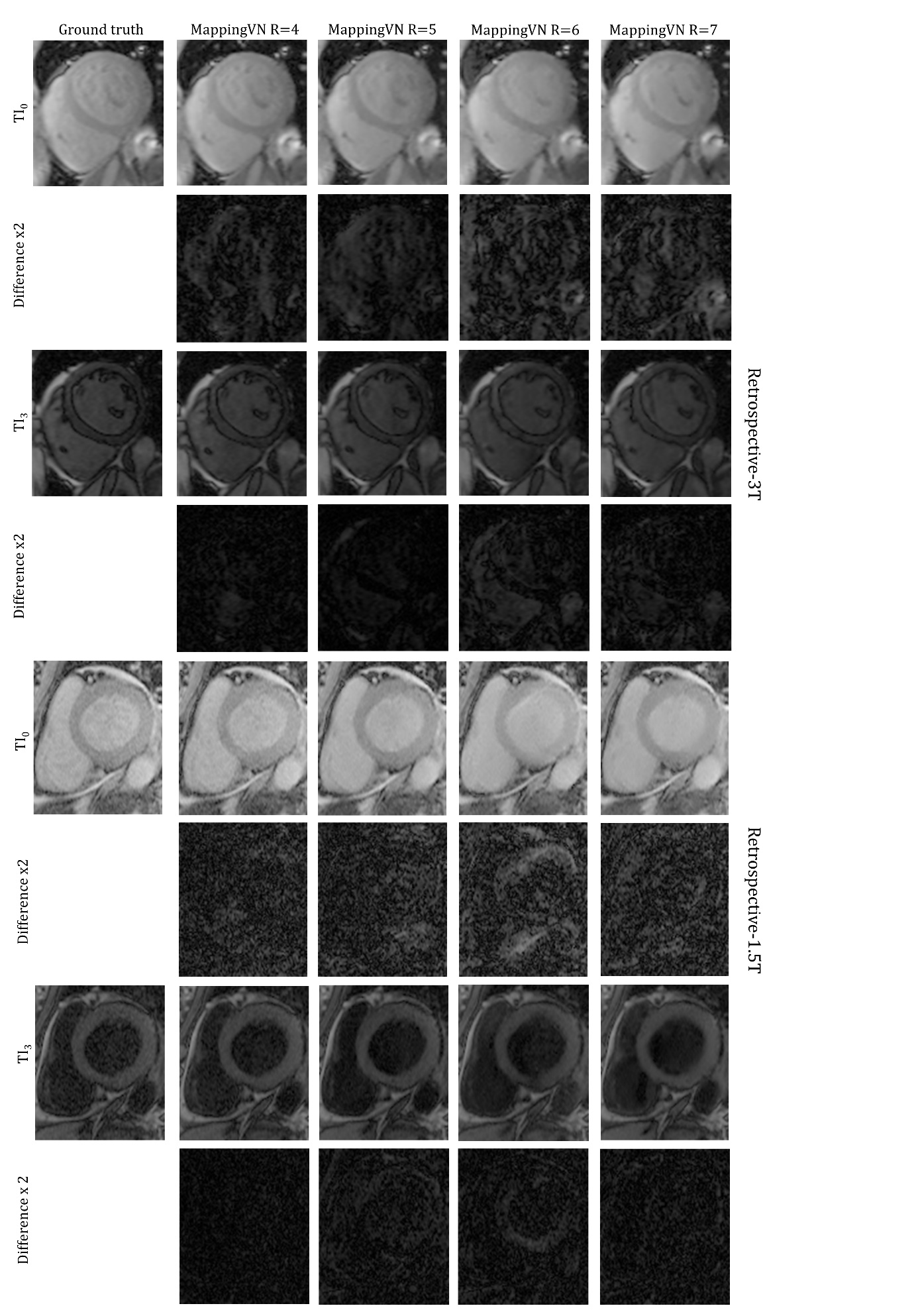


Supporting Information Figure S2: Example inversion recovery images retrospectively undersampled with acceleration rates ranging form 4 to 7 and reconstructed with MappingVN networks trained for the respective acceleration rate. Data from the Retrospective-3T and Retrospective-1.5T test sets was used. For each patient, the first and forth image of the re-ordered MOLLI images are shown. Percentile normalization was applied to improve the visibility, especially in the low signal images.


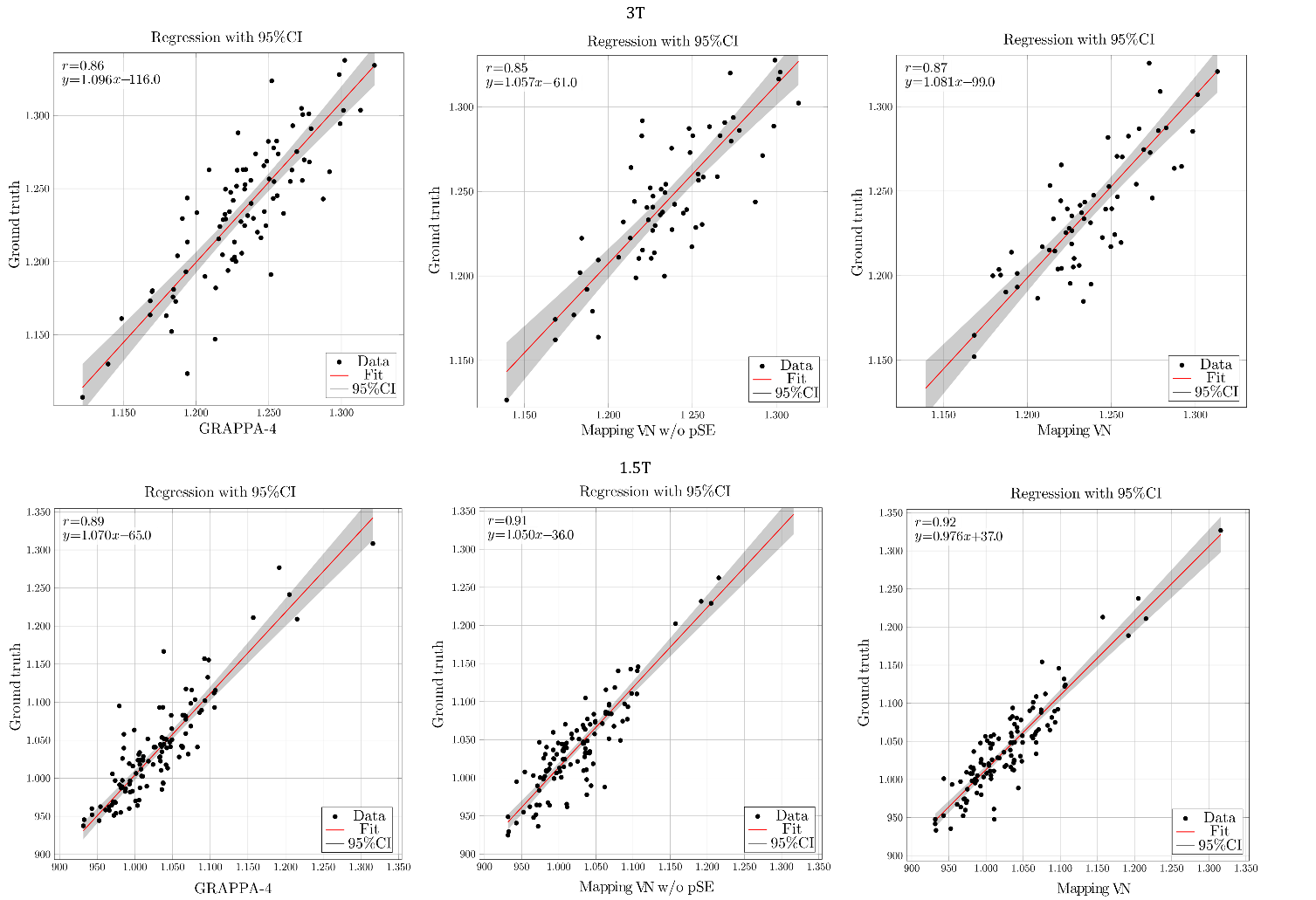


Supporting Information Figure S3: Regression analysis for the T1 comparisons performed using the Retrospective-3T and Retrospective-1.5T datasets. The plots show data points (black dots), the linear fit (red) and the corresponding 95% confidence intervals (gray) for the comparison of T1 maps reconstructed using GRAPPA-4 and the MappingVN with and without pSE layers to reference T1 maps. The slope and intercept values as well as the Pearson coefficient r are given in the top left corner of each subplot.


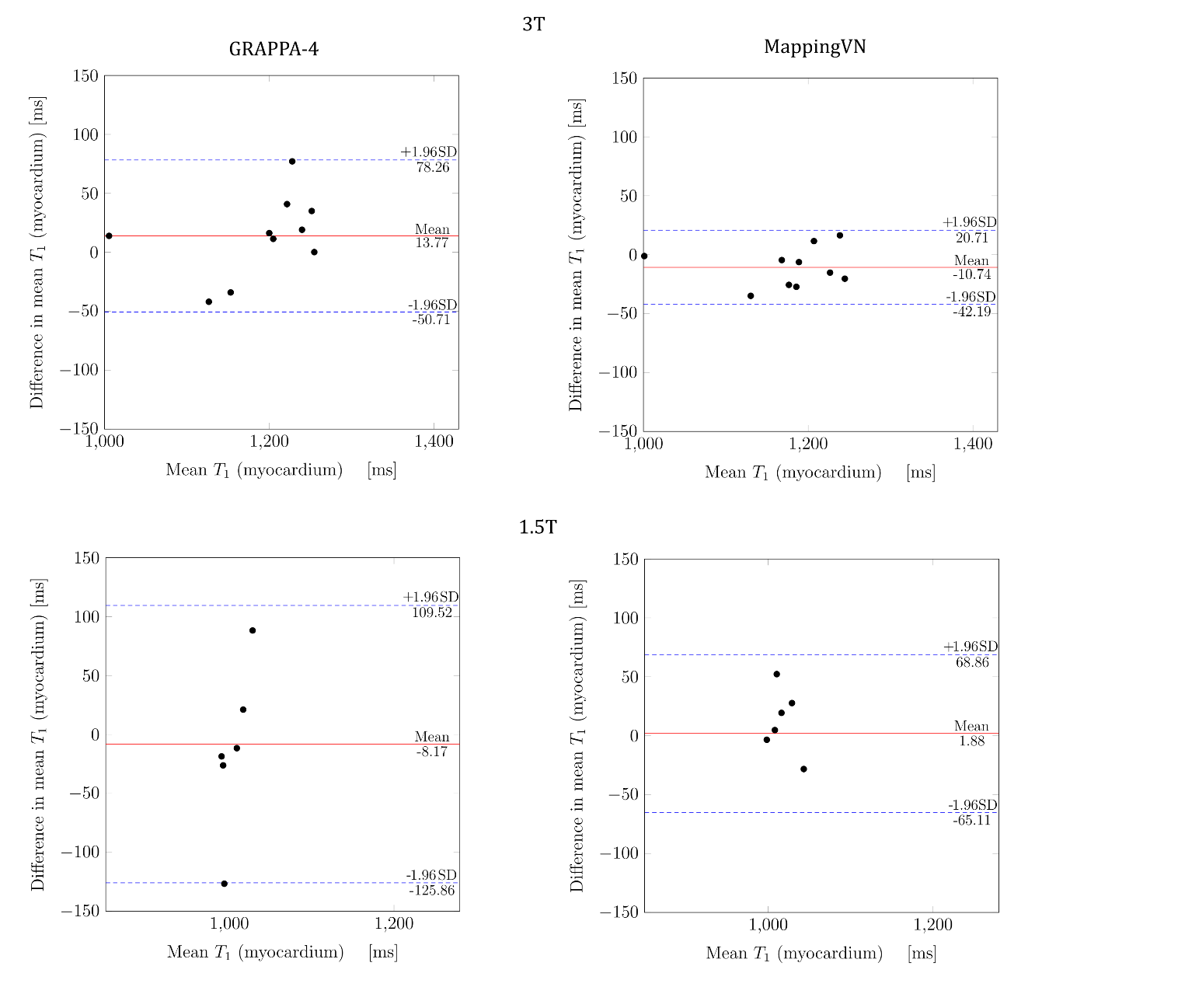


Supporting Information Figure S4: Result in prospective data in diastole for T1 agreement. Bland-Altman plots show the results for the evaluation of T1 agreement using GRAPPA-4 and the MappingVN in prospectively acquired high-resolution MOLLI acquisitions. Resulting T1 maps were compared to reference T1 maps in standard resolution, acquired in the same volunteer but in a different scan. Reference T1 values were subtracted from T1 values produced by the proposed method (proposed – reference).


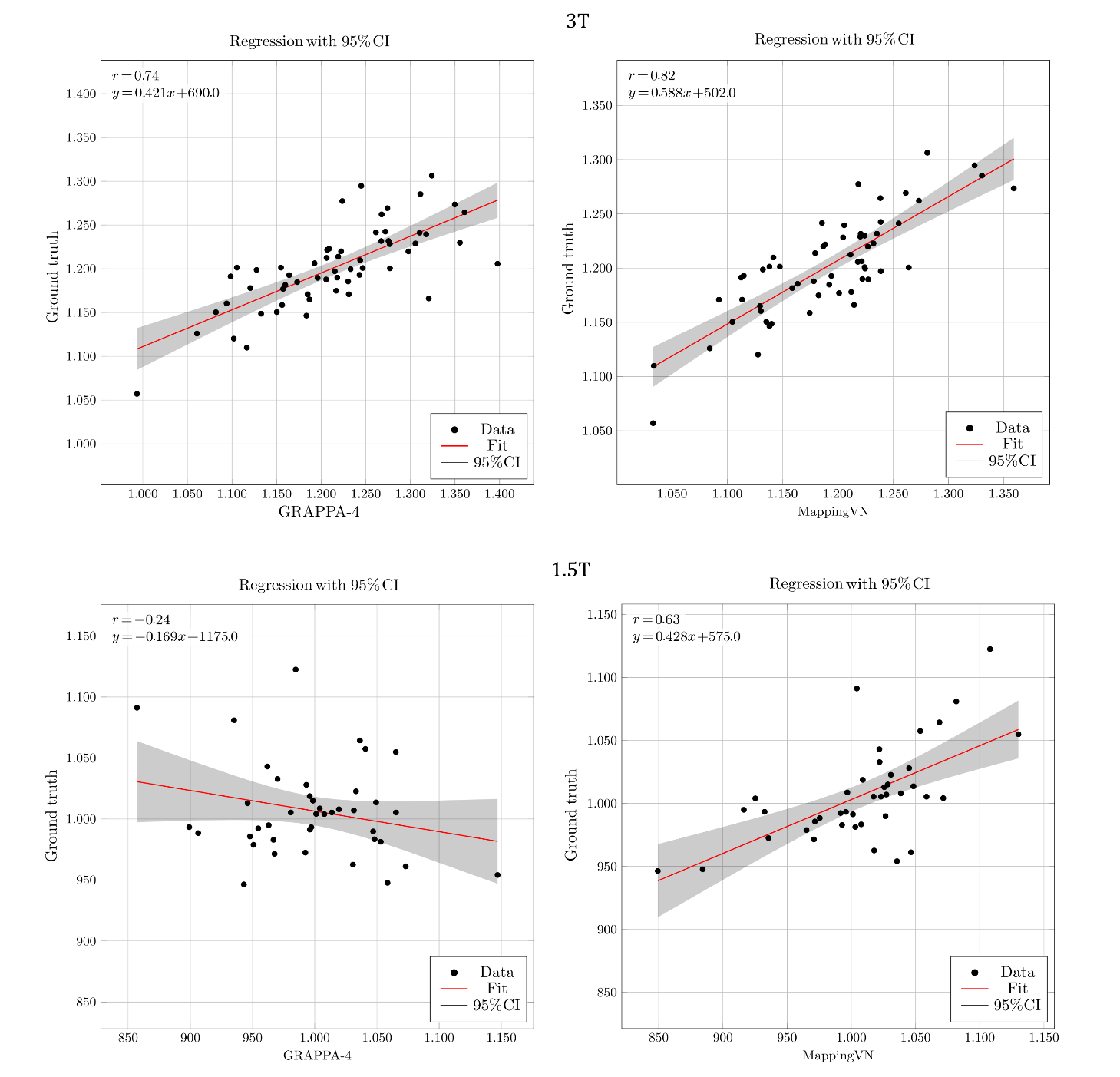


Supporting Information Figure S5: Regression analysis for the T1 comparisons performed using the Prospective-3T and Prospective-1.5T datasets. The plots show data points, the linear fit and the corresponding 95% confidence intervals for the comparison of high-resolution T1 maps reconstructed using GRAPPA-4 and the MappingVN to reference T1 maps in standard resolution. The slope and intercept values as well as the Pearson coefficient r are given in the top left corner of each subplot.


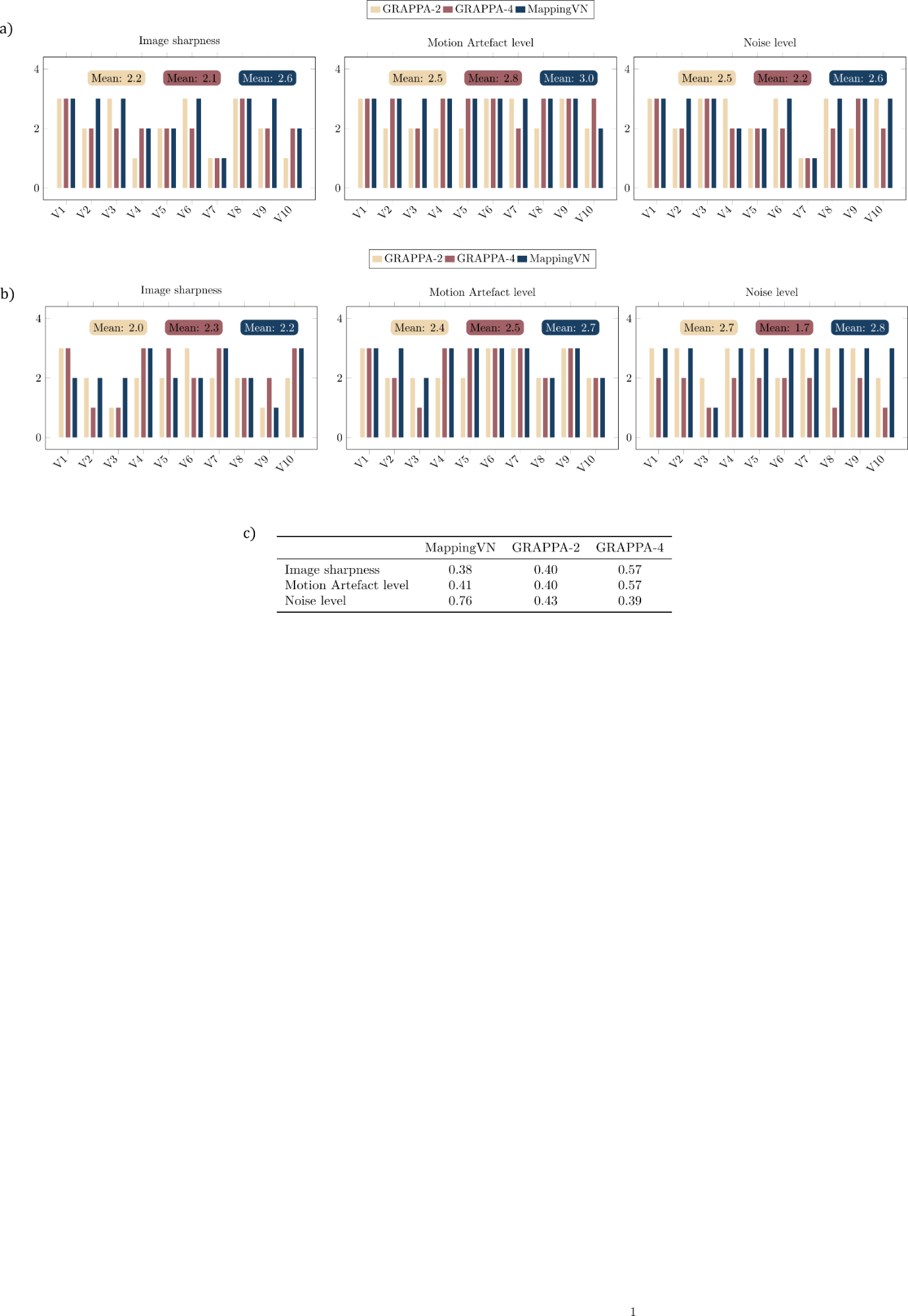


Supporting Information Figure S6: Results of the reader study for the systolic T1 mapping experiment, for (a) reader 1 and (b) reader 2. Corresponding mean scores are shown in color-coded boxes. The table in subfigure (c) shows corresponding coefficients computed using quadratically weighted Cohens Kappa.


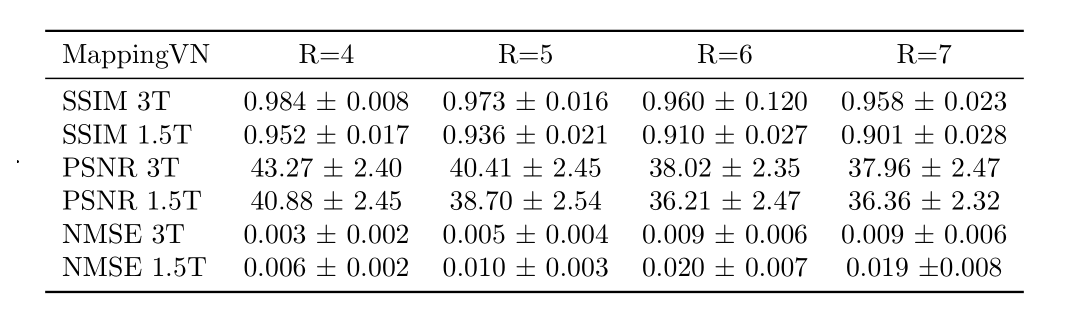


Supporting Information Table 1: Achieved metric scores when reconstructing retrospectively undersampled test data with acceleration rates between 4 and 7 using MappingVN networks trained for the respective acceleration rates.


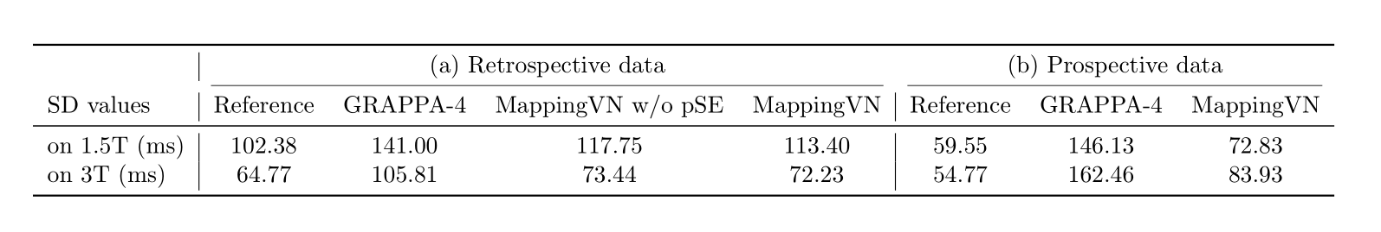


Supporting Information Table S2: Mean standard deviation (SD) values across the myocardium of T1 maps for all retrospective (a) and prospective (b) experiments. The mean SD is reported for all methods evaluated in terms of T1 accuracy.
